# Impact of Impaired Glucose Metabolism on Periodontitis Progression over Three Years

**DOI:** 10.3390/dj10010010

**Published:** 2022-01-07

**Authors:** Oelisoa M. Andriankaja, Kaumudi Joshipura, Francisco Muñoz, Bruce A. Dye, Frank B. Hu, Cynthia M. Pérez

**Affiliations:** 1 Center for Clinical Research and Health Promotion, School of Dental Medicine, Medical Sciences Campus, University of Puerto Rico, San Juan 00936-5067, Puerto Rico; kaumudi.joshipura@upr.edu (K.J.); fmunoz@upr.edu (F.M.); 2 Center for Oral Health Research, College of Dentistry, University of Kentucky, Lexington, KY 40536, USA; 3 Department of Epidemiology, Harvard T.H. Chan School of Public Health, Boston, MA 02115, USA; 4 National Institute of Dental and Craniofacial Research, Office of Science Policy and Analysis, National Institutes of Health, Bethesda, MD 20892, USA; bruce.dye@nih.gov; 5 Departments of Nutrition and Epidemiology, Harvard T.H. Chan School of Public Health, Boston, MA 02115, USA; frank.hu@channing.harvard.edu; 6 Channing Division of Network Medicine, Brigham and Women’s Hospital, Harvard Medical School, Boston, MA 02115, USA; 7 Department of Biostatistics and Epidemiology, Graduate School of Public Health, Medical Sciences Campus, University of Puerto Rico, San Juan 00936-5067, Puerto Rico; cynthia.perez1@upr.edu

**Keywords:** periodontal disease, pre-diabetic state, insulin resistance, obesity, cohort study

## Abstract

We evaluated the relationship between glucose abnormalities and periodontitis in overweight/obese individuals. Eight hundred and seventy (870) diabetes-free participants aged 40–65 years completed the three-year follow-up in the San Juan Overweight Adults Longitudinal Study. The ADA thresholds for fasting and 2-h post-load glucose and HbA1c were used to define prediabetes. The NHANES methods were used to assess periodontitis. Multivariable linear regression was used to evaluate the relationship between baseline glucose metabolism measures and periodontitis at follow-up, adjusting for potential confounders. There was no association between impaired glucose measures and mean pocket depth (PD), mean clinical attachment loss (CAL), or mean percent of sites ≥5 mm PD. Impaired glucose tolerance (IGT) was associated with a lower mean percent of sites ≥5 mm CAL (β = −1.6, *p* = 0.037). Prediabetes and impaired fasting glucose (IFG) were associated with improvement in mean percent of sites ≥5 mm PD (β = −1.4, *p* = 0.022; β = −1.6, *p* = 0.032; respectively). IFG and IGT were associated with improvement in mean percent of sites with ≥5 mm CAL (β = −1.6, *p* = 0.038; β = −1.9, *p* = 0.020; respectively). In conclusion, there were no consistent associations between baseline prediabetes or insulin resistance and periodontitis progression over a three-year period.

## 1. Introduction

The literature to date suggests that the relationship between periodontitis and diabetes is bidirectional [1]. Periodontal pathogens activate cytokines associated with increased levels of inflammatory markers and endothelial dysfunction, and altered lipid metabolism, which could lead to increased glucose abnormalities, insulin resistance, and increased risk of type 2 diabetes [1,2]. On the other hand, the hyperglycemic state in diabetes is associated with activated innate immunity, higher levels of inflammatory cytokines, expression of adhesion molecules, and production and accumulation of advanced glycation end products, which can contribute to periodontal tissue destruction [1,2]. 

A few large, longitudinal studies have evaluated the association between diabetes [3,4,5,6,7,8] and risk of periodontal disease and have found positive associations. Self-reported diabetes has also been associated with a significantly increased risk of self-reported periodontitis or tooth loss among male participants of the Health Professionals Follow-Up Study [6]. A systematic review of prospective cohort studies concluded that diabetes is associated with an increased risk of periodontitis onset or progression (RR = 1.86, 95% CI: 1.3–2.8) [9]. However, several methodological caveats were identified in these studies, including a short follow-up period, a small sample size, and the use of the Community Periodontal Index, which is a poor measure of periodontal disease.

Data relating pre-clinical stages of type 2 diabetes with periodontal disease have largely come from several cross-sectional studies [10,11,12,13,14,15,16,17,18,19]. Some cross-sectional studies have found no associations [10,20,21,22]. Only two large [3,23] and one small [24] longitudinal studies found associations between prediabetes and insulin resistance with periodontitis or gingival/periodontal inflammation. Three additional longitudinal studies [25,26,27] found that individuals with metabolic syndrome had a higher risk of incident or progression of periodontitis or tooth loss. However, Nascimento et al. [28] found no association between the metabolic syndrome components and several periodontitis measures. 

Given the inconsistent findings and limited longitudinal data, the San Juan Overweight Adults Longitudinal Study (SOALS) was initiated to improve our understanding of the relationship between precursors of type 2 diabetes and periodontitis, and to evaluate potential mediators of the relationship. 

## 2. Materials and Methods

### 2.1. Data Source and Study Population

**Figure 1:**
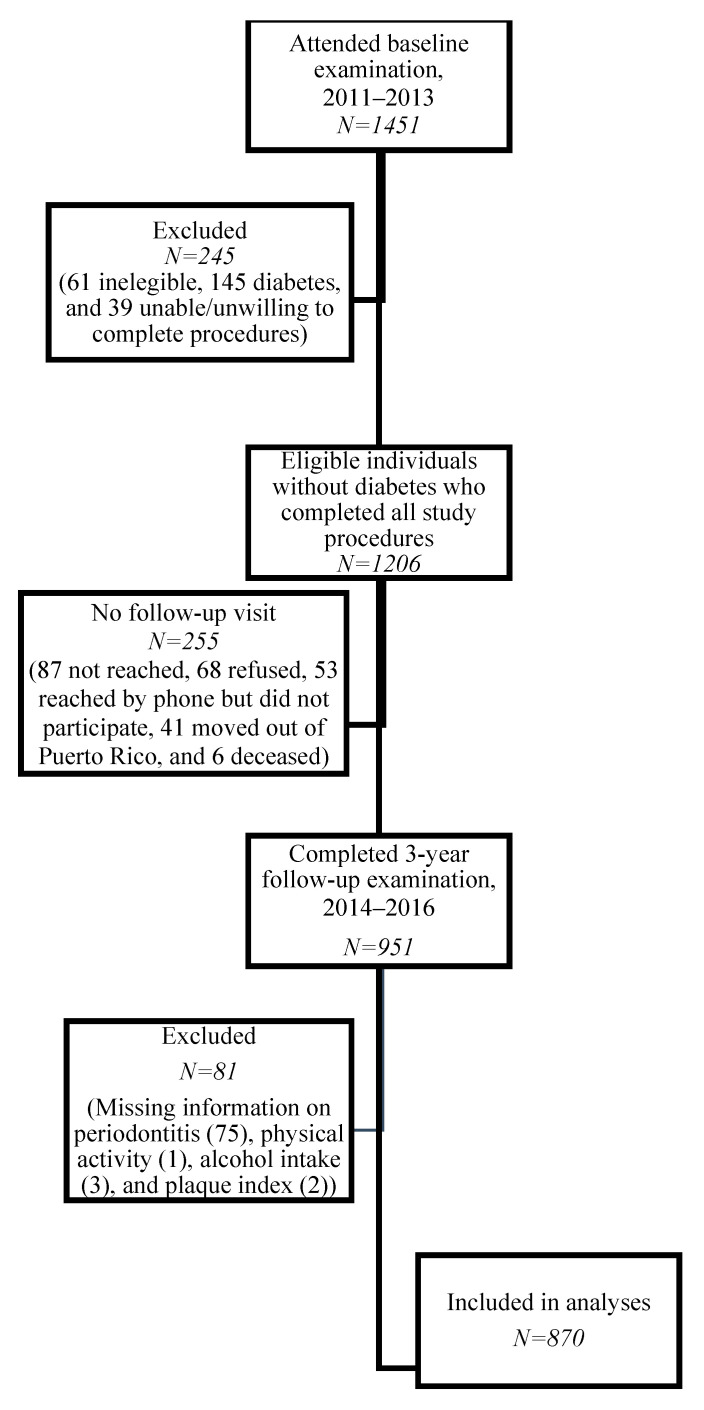
Flow of participants through the study (*N* = 870).

SOALS enrolled a convenience sample in 2011 and included evaluations at baseline and after a three-year follow-up period. Details about recruitment and retention have been previously reported [19,29,30]. Briefly, individuals aged 40–65 years who were overweight or obese (body mass index (BMI) ≥ 25 kg/m^2^), and free of clinically diagnosed diabetes were recruited from the San Juan municipality area in Puerto Rico. Exclusion criteria consisted mainly of factors precluding a valid periodontal exam (e.g., less than four teeth, orthodontic appliances) and factors that may put participants at risk (e.g., cardiovascular conditions and bleeding disorders). Out of the 1206 participants without diagnosed diabetes enrolled, 255 did not complete the follow-up. A total of 951 completed the follow-up visit (79% retention rate), of which 81 were further excluded for missing data; thus, our final analytical sample consisted of 870 participants (Figure 1). This study was approved by the human subject ethics board of the University of Puerto Rico Medical Sciences Campus [UPR Institutional Review Board (IRB), approved on 7 February 2010, IRB #A4840109] and was conducted in accordance with the Helsinki Declaration of 1975, as revised in 2013. Informed consent was obtained from all participants prior to performance of the study procedures. 

### 2.2. Definition of Periodontitis as Outcome of the Study 

Full mouth periodontal examinations were carried out at baseline (2011) and after three years of follow-up (2014). The National Health and Nutrition Examination Survey (NHANES) protocol for periodontitis assessment was used in both baseline and follow-up assessments [31,32] and performed by one of three calibrated dental examiners [19,29,30]. Periodontitis was assessed by clinical measurements of pocket depth (PD) and gingival recession at six sites per tooth for all teeth, except the third molars. PD was measured between the base of the sulcus or the pocket and the free gingival margin (FGM), and gingival recession was measured between the FGM and the cemento-enamel junction (CEJ) to compute the clinical attachment loss (CAL). Periodontitis was evaluated as mean PD, mean CAL, mean percentage of sites with PD ≥ 5 mm, and mean percentage of sites with CAL ≥ 5 mm at follow-up. Three-year changes in these measures were assessed as follow-up measure minus baseline measure, with positive values indicating worsening of PD and CAL and negative values indicating improvement over the three years. For quality assurance, 40 participants were evaluated by two of the three examiners at baseline and follow-up to assess reliability. Using information from all examiners, the pooled intra-class correlation coefficients using inter-proximal sites for mean CAL was 0.89 (95% CI: 0.81, 0.94) and for mean PD was 0.93 (95% CI: 0.88, 0.96). All examiners were in perfect agreement for the classification of the number of teeth. 

### 2.3. Definition of Baseline Glucose and Insulin Resistance Measures as Exposures in the Study

At baseline and follow-up exams, participants were asked to fast for 10 h before their appointment. Glucose and insulin levels were determined at fasting and 30, 60, and 120 min after administering a 75-g glucose load. Glucose was assessed using a Vitros System 250 instrument with an intra-assay coefficient of variation of 1.21% and inter-assay coefficient of variation of 3.06%. An immuno-enzymometric assay was used to determine plasma insulin using a TOSOH analyzer (intra-assay coefficient of variation = 1.49%; inter-assay coefficient of variation = 4.42%). HbA1c was assessed by a latex immunoagglutination inhibition methodology with monoclonal antibody using a Siemens Kit for DCA 2000 and DCA Vantage Analyzer. Using the American Diabetes Association (ADA) [33] criteria, participants without diagnosed diabetes mellitus who were not taking antihyperglycemic medications were classified as having type 2 diabetes mellitus if they had fasting glucose ≥126 mg/dL, 2hPG ≥ 200 mg/dL, or HbA1c ≥ 6.5%, or prediabetes if they had impaired fasting glucose (IFG, 100–125 mg/dL), impaired glucose tolerance (IGT, 140–199 mg/dL), or elevated HbA1c (5.7–6.4%). Participants were classified as having normal glycemia if all these values were below the mentioned thresholds for prediabetes^35^. The homeostatic model assessment of insulin resistance (HOMA-IR) was calculated as [fasting glucose (mg/dL) × fasting insulin (mg/dL)]/405]. Since there is no consensus on a cut point to define HOMA-IR, the study population specific 75th percentile was used (≥3.13).

### 2.4. Ascertainment of Covariates

In-person interviews and physical and laboratory assessments were performed. Information was collected on age, sex, education (less than high school, high school or more), smoking status (never, past, current), number of cigarettes per week, alcohol consumption (grams per week), frequency and type of physical activity during a typical week converted to the metabolic equivalent of task (MET) in hours per week, number of servings per week of fruits and vegetables, and family history of diabetes. Anthropometric measurements, including height, weight, and body circumferences (waist and hip), were taken 2–3 times according to the NHANES procedures and averaged [34]. The number of dental visits in the past year, frequency of tooth brushing and dental flossing, mouthwash use, and periodontal treatment during follow-up were recorded. The Silness and Loe Plaque Index, a measure of oral hygiene status, was assessed at six pre-selected teeth [35]. 

Blood pressure was taken three times after 1–2 min intervals [36], and the average was computed. Participants were classified as hypertensive (i.e., reported physician-diagnosed hypertension, currently taking antihypertensive medications, average SBP ≥ 140 mm Hg or average DBP ≥ 90 mm Hg at the baseline examination); pre-hypertensive (i.e., average SBP between 120- and 139-mm Hg or average DBP between 80- and 89-mm Hg); or normotensive (i.e., an average SBP < 120 mm Hg and average DBP < 80 mm Hg from the study assessment). High-density lipoprotein cholesterol (HDL-C) and triglycerides levels were assessed at a local laboratory reference using a commercially available enzymatic assay (Roche Diagnostics, Indianapolis, IN, USA).

### 2.5. Statistical Analysis

Baseline demographic and health characteristics were presented by glycemic status (normoglycemia and prediabetes). Separate multivariable linear regression models were used to examine the relationship between the exposure (prediabetes, IFG, IGT, elevated HbA1c, and HOMA-IR) at baseline, and each outcome measure (mean PD, mean CAL, mean percentage of sites with PD ≥ 5 mm, and mean percentage of sites with CAL ≥ 5 mm), while controlling for baseline periodontal measures. Similar analyses modeled three-year changes in mean PD, mean CAL, mean percentage of sites with PD ≥ 5 mm, mean percentage of sites with CAL ≥ 5 mm, and mean number of teeth as outcome measures. 

Baseline potential confounders were selected based on a priori knowledge and the underlying causal model of the hypothesized association between impaired glucose metabolism measures and periodontitis. The first multivariate model included age, sex, and smoking accounting for variation in follow-up time [37,38]. The second model additionally adjusted for education, physical activity, grams of alcohol consumption, waist circumference, fruit and vegetable intake, plaque index, and baseline periodontal measure (except for three-year changes in periodontal measures). The level of significance was set at *p* value < 0.05 and a 95% confidence interval (CI) was used for the statistical significance of the results. All statistical analyses were performed using Stata (StataCorp LP, College Station, TX, USA) for Windows version 15. 

## 3. Results

### 3.1. Baseline Characteristics of SOALS Participants According to Glycemic Status

The median follow-up for this cohort was 2.96 years (25th and 75th percentiles: 2.88 years and 3.01 years, respectively). Participants with prediabetes were more likely to be older (*p* < 0.001), less likely to be smokers (*p* = 0.012), had higher waist circumference (*p* < 0.001), triglycerides (*p* < 0.001), and glucose (*p* < 0.001), and had lower HDL-C (*p* = 0.013), and more moderate or severe periodontitis (*p* = 0.037), hypertension (*p* < 0.001), and insulin resistance (*p* < 0.001) compared to participants with normal glucose (Table 1).

### 3.2. Baseline and Three-Year Follow-Up Mean Pocket Depth and Mean Clinical Attachment Loss According to Impaired Glucose Metabolism Measures

Mean PD and mean CAL values were 2.1 ± 0.8 mm and 2.0 ± 1.3 mm, respectively, for those with prediabetes, and 2.0 ± 0.6 mm and 1.8 ± 1.1 mm, respectively, for those with normoglycemia (Table 2). There were slight improvements in PD and CAL between baseline and three-year follow-up in most subgroups defined by abnormal glucose measures (e.g., difference in mean PD in IFG: *p* = 0.041; difference in mean PD in HOMA-IR: *p* = 0.032). The mean percent of sites with ≥5 mm PD was slightly higher in individuals with prediabetes (4.2 ± 11.3%) than in those with normoglycemia (3.1 ± 7.8%) (data not shown). Similarly, the mean percent of sites with ≥5 mm CAL was higher (7.5 ± 15.5%) in individuals with prediabetes than those with normoglycemia (5.6 ± 11.7%). Changes in mean percent of sites with ≥5 mm PD and ≥5 mm CAL at baseline and follow-up suggest slight improvements in PD and CAL in individuals with abnormal glucose measures (data not shown).

### 3.3. Relationship between Baseline Glucose Metabolism Measures and Periodontitis Measures at Follow-Up

Linear regression models controlled for age, sex, smoking, education, physical activity, alcohol consumption, waist circumference, fruit and vegetable intake, plaque index, baseline periodontal measure, and follow-up time. Impaired glucose metabolism measures were not significantly associated with mean PD, mean CAL, and mean percent of sites with ≥5 mm PD at follow-up (Table 3). However, IGT was associated with a lower mean percent of sites with ≥5 mm CAL (β = −1.64, *p* = 0.037), while IFG was of borderline significance (β = −1.4, *p* = 0.063).

### 3.4. Relationship between Baseline Impaired Glucose Metabolism Measures with Three-Year Changes in Periodontitis Measures 

Changes in mean PD and mean CAL were not significantly associated with glucose metabolism measures (Table 4). However, both prediabetes and IFG were associated with improvement in mean percent of sites with ≥5 mm PD (β = −1.4, *p* = 0.02; β = −1.6, *p* = 0.032; respectively). Improvement in mean percent of sites with ≥5 mm CAL was associated with both IFG (β = −1.6, *p* = 0.038) and IGT (β = −1.9, *p* = 0.020) while its association with prediabetes was of borderline significance (β = −1.1, *p* = 0.078). Since severe periodontitis could lead to tooth loss, especially among individuals aged 40 years or over [39,40], the change in the mean number of teeth and incident tooth loss during the follow-up were also evaluated as secondary outcomes. Still, there were no significant associations (data not shown).

Further adjustment for other baseline factors, including visits to a dentist in the past year, frequency of tooth brushing or flossing, mouthwash use, blood pressure classification, number of teeth, medications use, and periodontal treatment during follow-up did not change the associations, and hence were not included in the final models. Subgroup analyses by smoking status and the number of teeth were conducted for each impaired glucose metabolism measure (data not shown). The directions of these associations were consistent across the teeth categories, and there was no apparent effect modification (data not shown). We also evaluated potential mediators for the associations where impaired glucose metabolism measures were significantly associated with periodontitis progression. Potential mediators (HDL-C, LDL-C, and triglycerides) were added to the final models one at a time, but the effect estimates were essentially similar and found no evidence of mediation by these factors (data not shown).

## 4. Discussion 

This longitudinal study is among the first to assess standard measures of impaired glucose metabolism and insulin resistance as risk factors for periodontitis onset or progression. This study does not corroborate a positive association between baseline prediabetes or insulin resistance and periodontitis progression over three years. These findings were also independent of major known confounders for the association between glucose metabolism and periodontitis, other than genetics, which is a limitation in most epidemiologic studies. The findings were also consistent among non-smokers, indicating that the results were not due to residual confounding by smoking. 

To date, there have only been a few longitudinal studies ascertaining whether hyperglycemia or insulin resistance is associated with increased risk of periodontal disease among people without diabetes, and some are limited by the diagnostic criterion used to assess periodontal disease. Chiu et al. [3] assessed the risk of prediabetes (fasting plasma glucose ≥100 mg/dL) in relation to periodontal disease, defined as a Community Periodontal Index score ≥ 3, over a five-year follow-up and found that participants with prediabetes had an elevated risk for periodontal disease (HR = 1.25; 95% CI: 1.00–1.57) after adjustment for confounders. However, the Community Periodontal Index does not assess recession or CAL as it is designed to assess PD at two different thresholds, underestimating periodontal status. Timonen et al. [24] examined the role of insulin resistance and altered beta-cell function on the four-year development of periodontal disease, defined by the presence of periodontal pockets (≥ 4 mm deep). However, the sample size was limited, PD was measured only at four pre-determined sites, and only the PD of the deepest site on each tooth was recorded, which may have underestimated disease severity. Our team assessed the role of insulin resistance on the risk of gingival/periodontal inflammation, as determined by the number of tooth sites with bleeding on probing (BOP) and number of teeth with PD ≥ 4 mm and BOP [23]. Participants in the highest HOMA-IR tertile at baseline had a significantly higher number of sites with BOP (RR = 1.19, 95% CI: 1.03–1.36) and number of teeth with PD ≥ 4 mm and BOP (RR = 1.39, 95% CI: 1.09–1.78) after comprehensive adjustment for confounders. Importantly this study assessed the number of sites with BOP since this measure indicates the presence of active status (or condition) of both reversible plaque-induced gingivitis and irreversible periodontitis. In contrast, the number of teeth with PD ≥ 4 mm and BOP reflects the number of teeth with a possible active status of periodontitis only [23]. Taken together, these studies support the need to elucidate the nature of the longitudinal association between impaired glucose metabolism and periodontitis.

However, our results were not consistent with our previous cross-sectional report in the same cohort, where we found that impaired glucose metabolism was significantly associated with severe periodontitis [19] and with other longitudinal studies showing similar positive associations [3,23,24]. This might be related to the slow rate of periodontal disease progression rendering to minimal or even no change of this oral disease within three years [40,41]. A longitudinal study conducted in a Swedish adult population estimated that the mean annual progression rate over three years was 0.06 mm for all individuals and varied between 0.04 and 0.07 mm per subject in the different age groups [41]. Whether the rate of periodontal disease progression over three years varies according to glycemic status is unclear. 

Strengths of the SOALS study are its longitudinal design, a relatively large sample size, and high-quality data. We used standard measures from fasting glucose, glucose tolerance tests, insulin, and HbA1c to evaluate precursors of diabetes. In addition, clinical assessment for periodontal measures was conducted using the NHANES oral health assessment protocol, the gold standard in clinical periodontal examinations, which included six sites around each tooth. We measured and adjusted for important potential confounders. 

Nonetheless, certain limitations need to be considered when interpreting these findings. Our population was limited to overweight and obese adults, and most participants in our study had at least a high school education, had a dental visit before the baseline visit, and reported treatment at baseline. These factors may have affected the generalizability of the findings. 

Since obesity is a significant risk factor for diabetes, limiting this high-risk group may be a possible reason for not seeing positive associations. However, no established biological pathways explain why the relationship between these two conditions could be in the opposite direction. We used a non-probability sampling to recruit the study group, limiting generalizability; however, the non-random sample would not affect the validity of the associations within this cohort. Moreover, due to logistical reasons, we could not use the same examiner at baseline and follow-up for many participants; this may have led to some random error or bias. 

One possible explanation for the inverse longitudinal associations observed in this study could be that participants who had impaired glucose metabolism at baseline were informed of their prediabetes and its potential association with the development of periodontitis, which may have motivated the adoption of healthy oral or other lifestyles. However, when we evaluated changes from baseline to follow-up data, people with prediabetes, compared to those with normoglycemia, did not show more frequent tooth brushing, flossing, periodontal treatment, dental visits in the past year, higher reduction in plaque scores, lipids, or inflammatory markers, nor a higher increase in physical activity over the follow-up period. These factors could reduce the risk of periodontitis. However, we did not evaluate changes in the oral microbiome, which could indicate a shift from more putative bacteria, causing an inflammatory response, to fewer putative bacteria.

In summary, this three-year follow-up study does not show consistent associations nor supports the hypothesis that prediabetes or IR is associated with PD progression in overweight/obese adults. In fact, the present study showed some non-significant improvement of pocket depth and significant improvement of clinical attachment loss rather than loosing attachment, which might have been related to change in lifestyles. Note, however, that PD may remain inactive or stable and without periodontal bleeding, while gingival recession continues to occur without being aware of it, which leads to continuous loss of attachment, and ultimately tooth loss.

Further research with larger samples and over more extended periods in different populations are warranted to understand the associations between glucose abnormalities and periodontitis.

## Figures and Tables

**Table 1:** Baseline characteristics of SOALS participants across glycemic status (n = 870) *.

	**Prediabetes** **(n = 492)**	**Normoglycemia** **(n = 378)**	***p*-Value**
Age (years)	51.6 ± 6.7	49.1 ± 6.6	<0.001
Male sex	26.6	26.7	0.975
High school education or more	90.5	89.4	0.616
Annual income ≥ $20,000	47.8	47.9	0.973
Smoking			0.012
Never	65.5	62.7	
Past smoker	18.3	14.3	
Current smoker	16.3	23.0	
Alcohol intake (grams/day)	2.2 ± 5.2	2.4 ± 6.5	0.585
Physical activity (METS/week)	22.5 ± 44.0	23.4 ± 37.8	0.754
Fruit and vegetable intake (servings/week)	7.0 ± 4.0	7.5 ± 4.1	0.084
Waist circumference (cm)	107.4 ± 14.5	103.9 ± 13.6	<0.001
BMI (kg/m^2^)	33.8 ± 6.3	32.5 ± 6.1	0.003
Plaque index	0.79 ± 0.60	0.81 ± 0.60	0.568
Periodontitis status			0.037
None/Mild	33.1	41.5	
Moderate	43.1	38.4	
Severe	23.8	20.1	
Number of teeth	23.4 ± 4.3	24.1 ± 4.1	0.026
Number of teeth in categories			0.216
25–28	48.8	55.3	
17–24	43.3	39.2	
11–16	6.3	4.2	
4–10	1.6	1.3	
Dentist visits in past 12 months	61.2	63.2	0.537
Tooth brushing more than once day	91.1	92.1	0.598
Dental flossing more than once a day	40.8	46.3	0.108
Any mouthwash use	51.8	55.0	0.349
Periodontal treatment	53.9	61.1	0.032
HDL-C (mg/dL)	47.2 ± 11.9	49.3 ± 13.1	0.013
Triglycerides (mg/dL)	157.9 ± 90.3	129.3 ± 58.2	<0.001
Blood pressure classification			<0.001
Normal	17.1	29.9	
Pre-hypertension	30.9	30.2	
Hypertension	52.0	40.0	
Fasting glucose (mg/dL)	96.3 ± 8.8	87.4 ± 5.8	<0.001
2-hr post load glucose (mg/dL)	124.5 ± 32.3	101.2 ± 19.6	<0.001
Fasting insulin (mIU/L)	12.1 ± 7.5	8.5 ± 5.1	<0.001
HbA1c (%)	5.9 ± 0.3	5.5 ± 0.2	<0.001
HOMA-IR	2.9 ± 1.9	1.9 ± 1.2	<0.001

* Values are presented as mean ± standard deviation or number (%). Abbreviations: BMI, Body mass Index; HDL-C, high density lipoprotein cholesterol; HbA1c, glycosylated hemoglobin; HOMA-IR, homeostatic model for insulin resistance.

**Table 2:** Mean levels of pocket depth (PD) and clinical attachment loss (CAL) at baseline and follow-up visits according to impaired glucose metabolism measures.

	Mean PD (mm)			Mean CAL (mm)		
	Baseline	Follow-Up	*p*-Value	Baseline	Follow-Up	*p*-Value
Prediabetes			0.143			0.553
Yes (n = 492)	2.1 ± 0.8	2.0 ± 0.7		2.0 ± 1.3	1.9 ± 1.2	
No (n = 378)	2.0 ± 0.6	2.0 ± 0.7		1.8 ± 1.1	1.8 ± 1.2	
	*p* = 0.223	*p* = 0.995		*p* = 0.042	*p* = 0.105	
IFG			0.041			0.161
Yes (n = 183)	2.2 ± 0.8	2.0 ± 0.7		2.1 ± 1.3	2.0 ± 1.2	
No (n = 687)	2.0 ± 0.7	2.0 ± 0.7		1.9 ± 1.2	1.9 ± 1.2	
	*p* = 0.020	*p* = 0.494		*p* = 0.010	*p* = 0.110	
IGT			0.202			0.139
Yes (n = 156)	2.1 ± 0.7	2.0 ± 0.6		2.0 ± 1.1	1.8 ± 0.9	
No (n = 714)	2.1 ± 0.7	2.0 ± 0.7		1.9 ± 1.2	1.9 ± 1.2	
	*p* = 382	*p* = 0.846		*p* = 0.600	*p* = 0.601	
Elevated HbA1c			0.78			0.706
Yes (n = 496)	2.1 ± 0.8	2.0 ± 0.7		2.0 ± 1.2	1.9 ± 1.2	
No (n = 374)	2.1 ± 0.7	2.0 ± 0.7		1.9 ± 1.1	1.8 ± 1.2	
	*p* = 0.610	*p* = 0.441		*p* = 0.273	*p* = 0.171	
HOMA-IR			0.032			0.196
Yes (n = 189)	2.2 ± 0.9	2.1 ± 0.7		2.1 ± 1.3	2.0 ± 1.2	
No (n = 681)	2.0 ± 0.7	2.0 ± 0.7		1.9 ± 1.2	1.9 ± 1.2	
	*p* < 0.001	*p* = 0.030		*p* = 0.017	*p* = 0.136	

Note: Prediabetes was defined as impaired fasting glucose (IFG, 100–125 mg/dL), impaired glucose tolerance (IGT, 140–199 mg/dL), or elevated HbA1c (HbA1c, 5.7–6.4%)]. HOMA-IR was defined based on the study population-specific 75th percentile (≥3.1). Abbreviations: IFG: Impaired fasting glucose; IGT: 2hr-Impaired Glucose tolerance; HbA1c, glycosylated hemoglobin; HOMA-IR, homeostatic model for insulin resistance.

**Table 3:** Multivariable linear regression models for the relationship between baseline glucose metabolism measures and periodontitis measures at follow-up.

	Mean PD, mm		Mean CAL, mm		Mean Percent of Sites with ≥5 mm PD		Mean Percent of Sites with ≥5 mm CAL	
	β (SE)	*p* Value	β (SE)	*p* Value	β (SE)	*p* Value	β (SE)	*p* Value
Prediabetes								
Model 1	0.0 (0.0)	0.995	0.1 (0.1)	0.105	−0.1 (0.6)	0.81	0.8 (1.0)	0.424
Model 2	0.0 (0.0)	0.645	0.0 (0.1)	0.761	−0.6 (0.5)	0.244	−0.5 (0.6)	0.422
Model 3	0.0 (0.0)	0.704	0.0 (0.1)	0.97	−0.5 (0.5)	0.3	−0.8 (0.6)	0.203
IFG								
Model 1	0.0 (0.1)	0.494	0.2 (0.1)	0.11	0.1 (0.7)	0.919	0.5 (1.2)	0.7
Model 2	0.0 (0.0)	0.309	0.0 (0.1)	0.577	−0.8 (0.6)	0.158	−1.5 (0.8)	0.057
Model 3	0.0 (0.1)	0.314	0.0 (0.1)	0.595	−0.8 (0.6)	0.164	−1.4 (0.8)	0.063
IGT								
Model 1	0.0 (0.1)	0.846	−0.1 (0.1)	0.6	−0.3 (0.7)	0.679	−1.7 (1.3)	0.192
Model 2	0.0 (0.0)	0.522	−0.1 (0.1)	0.324	−0.4 (0.6)	0.525	−1.9 (0.8)	0.016
Model 3	0.0 (0.0)	0.585	0.0 (0.1)	0.534	−0.3 (0.6)	0.593	−1.6 (0.8)	0.037
High HbA1c								
Model 1	0.0 (0.0)	0.441	0.1 (0.1)	0.171	−0.8 (0.6)	0.149	0.9 (1.0)	0.379
Model 2	0.0 (0.0)	0.963	0.0 (0.1)	0.385	−0.6 (0.5)	0.221	0.2 (0.6)	0.702
Model 3	0.0 (0.0)	0.822	0.0 (0.1)	0.711	−0.5 (0.5)	0.286	−0.2 (0.6)	0.754
HOMA-IR								
Model 1	0.1 (0.1)	0.03	0.1 (0.1)	0.136	0.7 (0.7)	0.307	1.6 (1.2)	0.182
Model 2	0.0 (0.0)	0.622	0.0 (0.1)	0.833	−0.4 (0.6)	0.521	−0.3 (0.7)	0.655
Model 3	0.0 (0.0)	0.708	0.0 (0.1)	0.915	−0.3 (0.6)	0.637	−0.4 (0.8)	0.589

Note: The reference category for prediabetes, IFG, and IGT is normal glycemia; for elevated HbA1c is normal HbA1c; and for HOMA-IR is the lower three quartiles combined. Model 1: unadjusted model; Model 2 adjusted for age, sex, smoking, and follow-up time; Model 3 additionally adjusted for education, physical activity (METs), alcohol consumption (grams/week), waist circumference, fruit and vegetable intake, total number of teeth, plaque index, and baseline periodontal measure. Abbreviations: IFG: Impaired fasting glucose; IGT: 2hr-Impaired Glucose tolerance; HbA1c, glycosylated hemoglobin; HOMA-IR, homeostatic model for insulin resistance.

**Table 4:** Results from multivariable linear regression models for the relationship between glucose metabolism measures and three-year changes in periodontitis.

	Change in Mean PD (mm)		Change in Mean CAL (mm)		Change in Mean Percent of Sites with ≥5 mm PD		Change in Mean Percent of Sites with ≥5 mm CAL	
	β (SE)	*p* Value	β (SE)	*p* Value	β (SE)	*p* Value	β (SE)	*p* Value
Prediabetes								
Model 1	−0.1 (0.0)	0.143	0.0 (0.1)	0.553	−1.3 (0.6)	0.029	−1.1 (0.6)	0.104
Model 2	−0.1 (0.0)	0.167	0.0 (0.1)	0.685	−1.4 (0.6)	0.022	−0.8 (0.7)	0.197
Model 3	−0.1 (0.0)	0.177	0.0 (0.1)	0.465	−1.4 (0.6)	0.022	−1.1 (0.6)	0.078
IFG								
Model 1	−0.1 (0.1)	0.041	−0.1 (0.1)	0.161	−1.7 (0.7)	0.018	−1.9 (0.8)	0.013
Model 2	−0.1 (0.1)	0.071	−0.1 (0.1)	0.337	−1.7 (0.8)	0.025	−1.7 (0.8)	0.031
Model 3	−0.1 (0.1)	0.097	−0.1 (0.1)	0.387	−1.6 (0.7)	0.032	−1.6 (0.8)	0.038
IGT								
Model 1	−0.1 (0.1)	0.202	−0.1 (0.1)	0.139	−0.8 (0.8)	0.323	−2.5 (0.8)	0.003
Model 2	−0.1 (0.1)	0.23	−0.1 (0.1)	0.244	−0.8 (0.8)	0.313	−2.1 (0.8)	0.011
Model 3	−0.1 (0.1)	0.298	−0.1 (0.1)	0.332	−0.7 (0.8)	0.387	−1.9 (0.8)	0.02
High HbA1c								
Model 1	0.0 (0.0)	0.8	0.0 (0.1)	0.706	−0.6 (0.6)	0.299	0.0 (0.6)	0.992
Model 2	0.0 (0.0)	0.801	0.0 (0.1)	0.727	−0.8 (0.6)	0.214	0.0 (0.7)	0.943
Model 3	0.0 (0.0)	0.813	0.0 (0.1)	0.877	−0.8 (0.6)	0.204	−0.4 (0.7)	0.528
HOMA-IR								
Model 1	−0.1 (0.0)	0.032	−0.1 (0.1)	0.196	−1.4 (0.7)	0.044	−1.0 (0.8)	0.216
Model 2	−0.1 (0.0)	0.031	−0.1 (0.1)	0.225	−1.4 (0.7)	0.056	−0.8 (0.8)	0.273
Model 3	−0.1 (0.1)	0.128	−0.1 (0.1)	0.316	−1.0 (0.8)	0.168	−0.9 (0.8)	0.251

Note: The reference category for prediabetes, IFG, and IGT is normal glycemia; for elevated HbA1c is normal HbA1c; and for HOMA-IR is the lower three quartiles combined. Model 1: unadjusted model; Model 2 adjusted for age, sex, smoking, and follow-up time; Model 3 additionally adjusted for education, physical activity (METs), alcohol consumption (grams/week), waist circumference, fruit and vegetable intake (servings/week), total number of teeth and plaque index. Abbreviations: IFG: Impaired fasting glucose; IGT: 2hr-Impaired Glucose tolerance; HbA1c, glycosylated hemoglobin; HOMA-IR, homeostatic model for insulin resistance.

## Data Availability

The data presented in this study are available on request from the co-author, Kaumudi Joshipura (kaumudi.joshipura@upr.edu). The data are not publicly available due to privacy and ethical restrictions.
